# Supplementary material for: Leveraging the genetic diversity of trout in the rivers of the British Isles and northern France to understand the movements of sea trout (Salmo trutta L.) around the English Channel
Source: Evol Appl. 2024 Jul 22;17(7):e13759. doi: 10.1111/eva.13759 (PMC11261213; doi:10.1111/eva.13759)
Supplement: Supplementary file 2 — Figures S1–S5 [file EVA-17-e13759-s003.docx]

**Supplementary Figures for:**

**Leveraging the genetic diversity of trout in the rivers of the southern British Isles and northern France to understand the movements of sea trout (*Salmo trutta* L.) around the English Channel**

**Page 2 –** Supplementary Figure 1 – map of marine and estuarine sampling locations

**Page 3** – Supplementary Figure 2 – *RUBIAS* baseline group-level Leave-One-Out accuracy and efficiency

**Page 4** – Supplementary Figure 3 – *RUBIAS* baseline river-level Leave-One-Out accuracy and efficiency

**Page 5** – Supplementary Figure 4 – Comparison of mixed stock and individual assignment analyses for cBayes and *RUBIAS* for known-origin trout

**Page 6** – Supplementary Figure 5 – Evanno et al. (2005) delta *K* (Δ*K* ) results for the hierarchical STRUCTURE analyses of genetic structuring

**Supplementary Figure 1** Map giving the approximate location for eight marine (white label) and four estuarine (grey label) collections of anadromous trout. Marine collection abbreviations: COR – southern Cornwall; KIM – Kimmeridge Bay; RYE – Rye Harbour; EAN – East Anglian drift-net fishery; SAA – Saâne illegal nets; CRI – Criel-sur-Mer recreational beach nets; MER – Mers-les-Bains and Le Tréport recreational beach nets; DUT – Dutch commercial fishery by-catch. Estuarine collection abbreviations: TT – Taw/Torridge shared estuary; TAM – River Tamar tidal limit fish trap; PLH – Poole Harbour; OUS – Sussex Ouse estuary recreational rod fishery

**
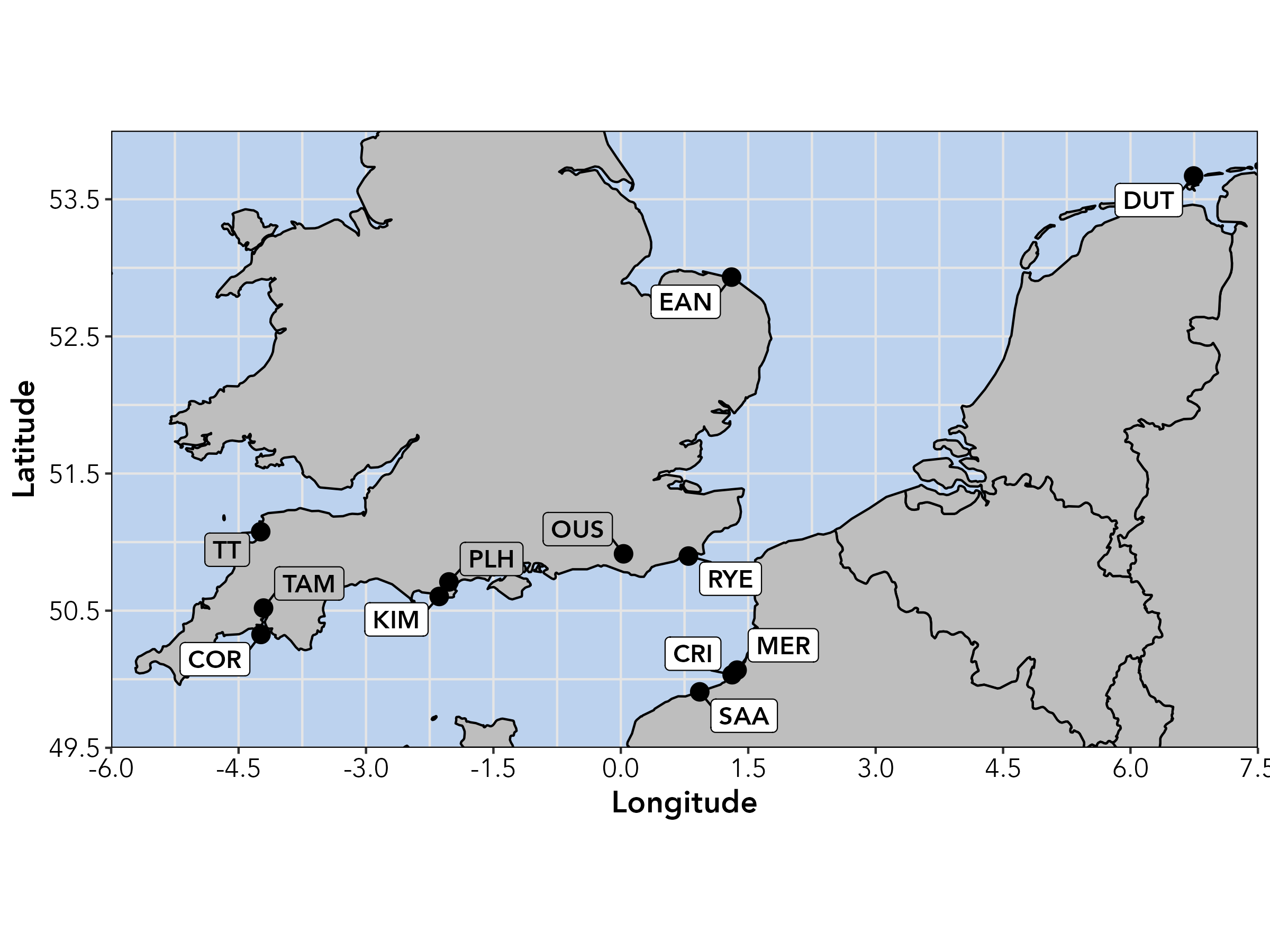
**

**Supplementary Figure 2** *RUBIAS* group-level Leave-One-Out accuracy (red) and efficiency (blue). Reporting group codes are SEIRE – south east Ireland, OUTBRCH – outer Bristol Channel, INNBRCH – inner Bristol Channel, DEVCORN – Devon & Cornwall, LANDSEND – Land’s End, HANTS – Hampshire Basin, SEENG – south east England, THAMESEA – Thames & East Anglia, NEENG – north east England, BRET – Bretagne, LOWNORM – Lower Normandie, UPPNORM – Upper Normandie, DENMARK – Denmark and FRHAT – French hatcheries.


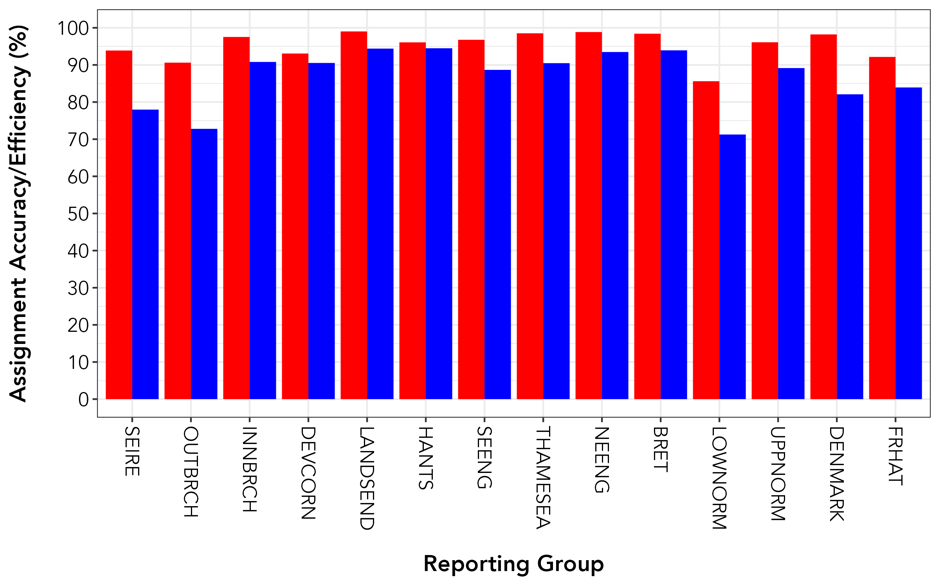


**Supplementary Figure 3** *RUBIAS* river-level Leave-One-Out accuracy (red) and efficiency (blue). River codes are as given in Table X. Reporting group codes are SEIRE – south east Ireland, OUTBRCH – outer Bristol Channel, INNBRCH – inner Bristol Channel, DEVCORN – Devon & Cornwall, LANDSEND – Land’s End, HANTS – Hampshire Basin, SEENG – south east England, THAMESEA – Thames & East Anglia, NEENG – north east England, BRET – Bretagne, LOWNORM – Lower Normandie, UPPNORM – Upper Normandie, DENMARK – Denmark and FRHAT – French hatcheries. For clarity, the Devon & Cornwall rivers have been splitting into two groups.


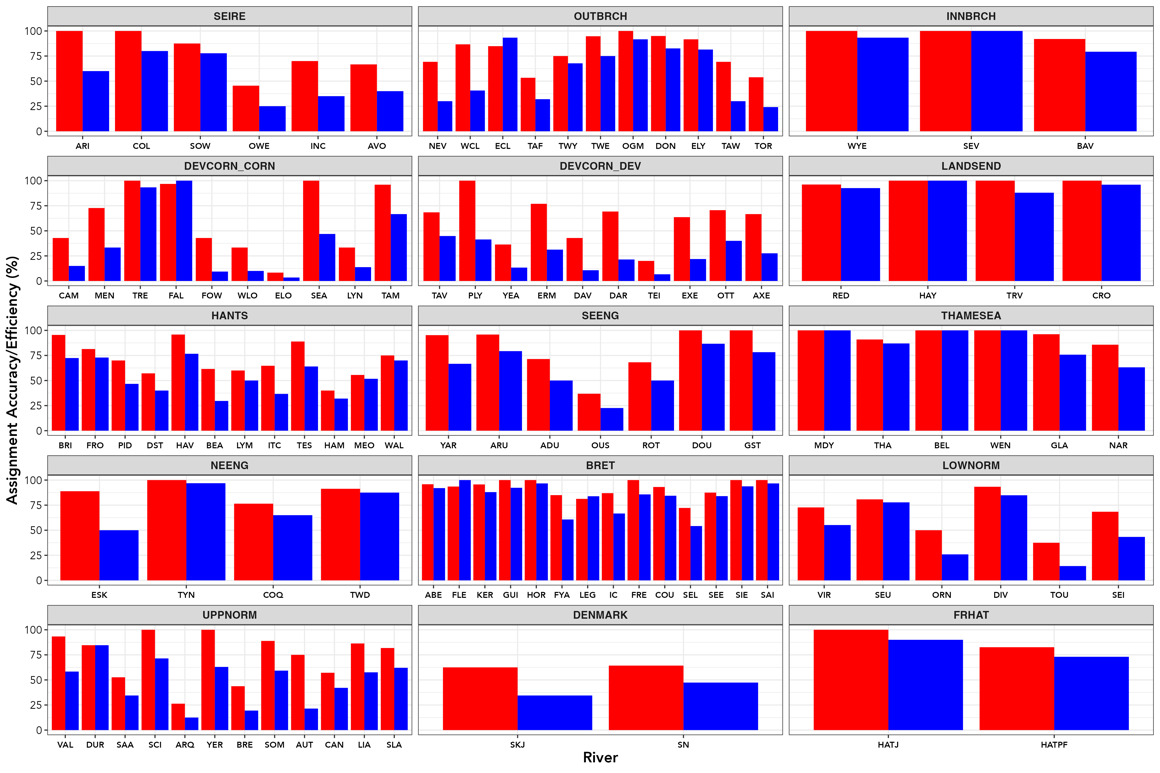


**Supplementary Figure 4** Comparison of mixed stock (MSA) and individual assignment (IA) analyses as performed in cBayes (red) and *RUBIAS* (blue) for known-origin samples of trout from 25 baseline rivers to reporting group and river of origin. River codes are as given in Supplementary Table 1. Assignment proportions are presented for the MSA and proportion of correct assignments, assuming an assignment probability ≥ 0.7 for IA.


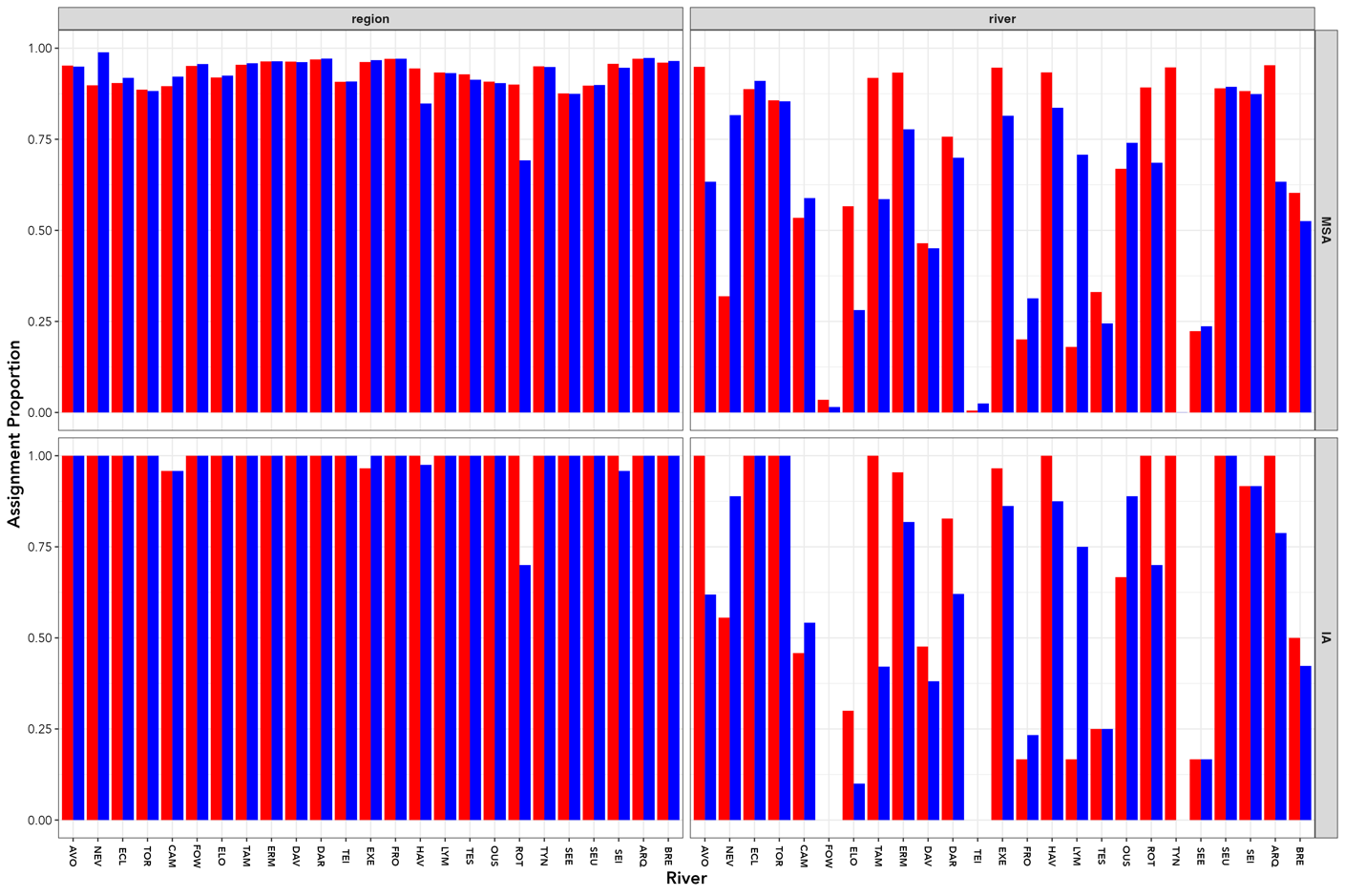


**Supplementary Figure 5** Results for Evanno et al. (2005) delta *K* (Δ*K* ) for the hierarchical STRUCTURE analyses of genetic structuring in English Channel brown trout populations.

**
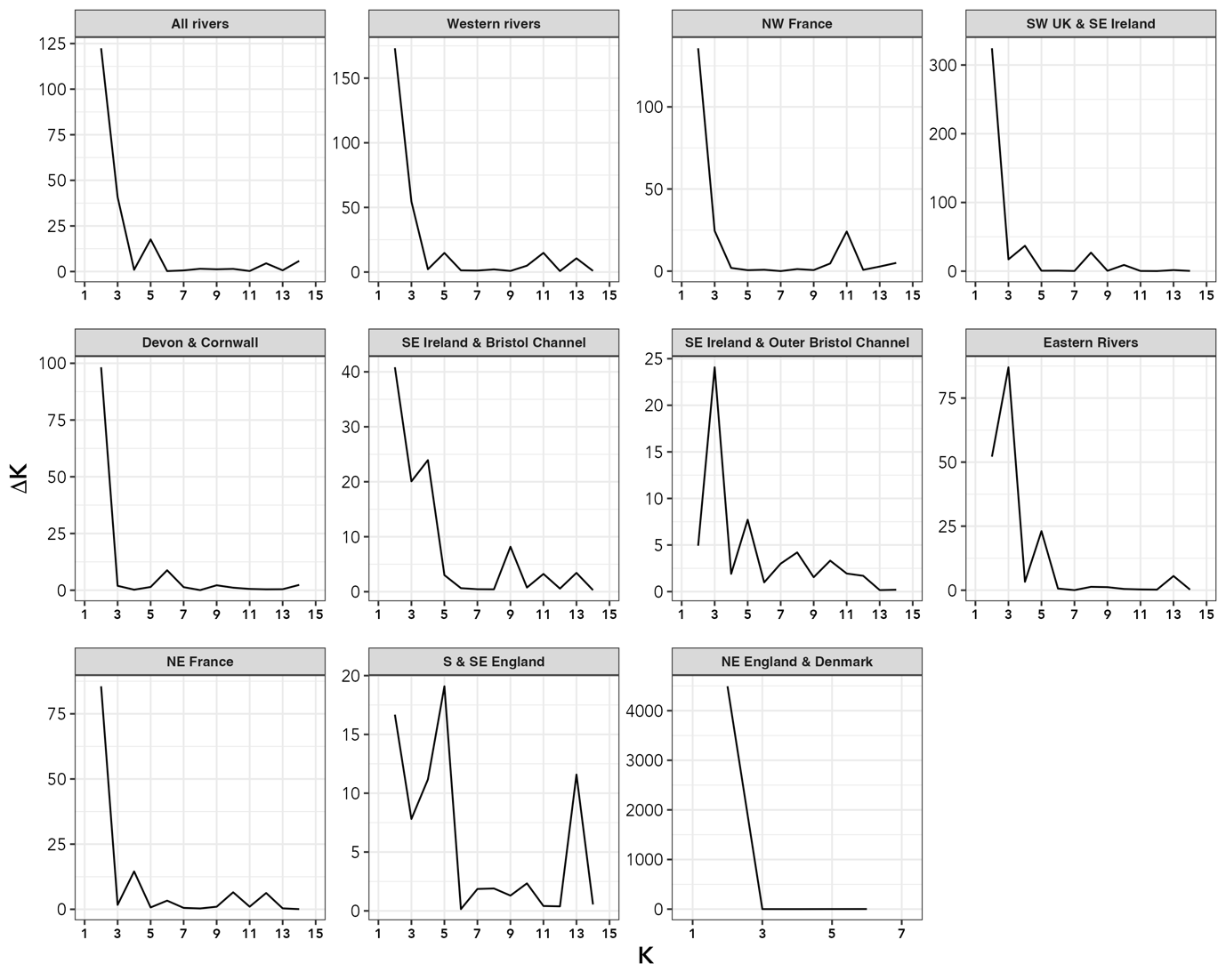
**
