# Supplementary material for: Leveraging the genetic diversity of trout in the rivers of the British Isles and northern France to understand the movements of sea trout (Salmo trutta L.) around the English Channel
Source: Evol Appl. 2024 Jul 22;17(7):e13759. doi: 10.1111/eva.13759 (PMC11261213; doi:10.1111/eva.13759)
Supplement: Supplementary file 3 — Table S1 [file EVA-17-e13759-s007.docx]

**Supplementary Table for:**

**Leveraging the genetic diversity of trout in the rivers of the southern British Isles and northern France to understand the movements of sea trout (*Salmo trutta* L.) around the English Channel**

**Supplementary Table 1** – Details of rivers sampled for resident brown trout for the construction of the single nucleotide polymorphism baseline. Latitude and longitude data are given for the mouth of each river.

| **River** | **Code** | **Country** | **Reporting Group** | **Reporting Group Code** | **Latitude** | **Longitude** | **Number genotyped** | **Removed Full sibs** | **Failed samples** | **Baseline samples** | **Known origin samples** |
| --- | --- | --- | --- | --- | --- | --- | --- | --- | --- | --- | --- |
| Arigideen | ARI | Ireland | South east Ireland | SEIRE | 51.625845 | -8.668346 | 20 | 0 | 0 | 20 | 0 |
| Colligan | COL | Ireland | South east Ireland | SEIRE | 52.071162 | -7.513121 | 20 | 0 | 0 | 20 | 0 |
| Sow | SOW | Ireland | South east Ireland | SEIRE | 52.316017 | -6.363038 | 20 | 2 | 0 | 18 | 0 |
| Owenavaragh | QWE | Ireland | South east Ireland | SEIRE | 52.654237 | -6.220131 | 20 | 0 | 0 | 20 | 0 |
| Inch | INC | Ireland | South east Ireland | SEIRE | 52.711022 | -6.166956 | 20 | 0 | 0 | 20 | 0 |
| Avoca | AVO | Ireland | South east Ireland | SEIRE | 52.793996 | -6.135749 | 41 | 0 | 0 | 20 | 21 |
| Nevern | NEV | Wales | Outer Bristol Channel | OUTBRCH | 52.026942 | -4.85074 | 40 | 0 | 1 | 30 | 9 |
| West Cleddau | WCL | Wales | Outer Bristol Channel | OUTBRCH | 51.663269 | -5.154108 | 32 | 0 | 0 | 32 | 0 |
| East Cleddau | ECL | Wales | Outer Bristol Channel | OUTBRCH | 51.663269 | -5.154108 | 40 | 0 | 0 | 30 | 10 |
| Taff | TAF | Wales | Outer Bristol Channel | OUTBRCH | 51.7168 | -4.41441 | 30 | 5 | 0 | 25 | 0 |
| Twyi | TWY | Wales | Outer Bristol Channel | OUTBRCH | 51.7168 | -4.41441 | 32 | 0 | 1 | 31 | 0 |
| Tawe | TWE | Wales | Outer Bristol Channel | OUTBRCH | 51.610081 | -3.927836 | 30 | 5 | 1 | 24 | 0 |
| Ogmore | OGM | Wales | Outer Bristol Channel | OUTBRCH | 51.468126 | -3.645016 | 24 | 0 | 0 | 24 | 0 |
| Wye | WYE | Wales | Inner Bristol Channel | INNBRCH | 51.608194 | -2.662412 | 30 | 0 | 0 | 30 | 0 |
| Severn | SEV | England | Inner Bristol Channel | INNBRCH | 51.657636 | -2.589118 | 29 | 0 | 1 | 28 | 0 |
| Bristol Avon | BAV | England | Inner Bristol Channel | INNBRCH | 51.506059 | -2.726962 | 30 | 1 | 0 | 29 | 0 |
| Doniford | DON | England | Outer Bristol Channel | OUTBRCH | 51.184262 | -3.304221 | 24 | 1 | 0 | 23 | 0 |
| East Lyn | ELY | England | Outer Bristol Channel | OUTBRCH | 51.23501 | -3.829387 | 30 | 3 | 0 | 27 | 0 |
| Taw | TAW | England | Outer Bristol Channel | OUTBRCH | 51.07585 | -4.239722 | 30 | 0 | 0 | 30 | 0 |
| Torridge | TOR | England | Outer Bristol Channel | OUTBRCH | 51.07585 | -4.239722 | 38 | 0 | 1 | 29 | 8 |
| Camel | CAM | England | Devon & Cornwall | DEVCORN | 50.577934 | -4.948684 | 64 | 0 | 0 | 40 | 24 |
| Menalhyl | MEN | England | Devon & Cornwall | DEVCORN | 50.468438 | -5.039764 | 24 | 0 | 0 | 24 | 0 |
| Red River | RED | England | Land's End | LANDSEND | 50.230971 | -5.392895 | 30 | 0 | 3 | 27 | 0 |
| Hayle | HAY | England | Land's End | LANDSEND | 50.200946 | -5.437558 | 32 | 2 | 0 | 30 | 0 |
| Trevaylor | TRV | England | Land's End | LANDSEND | 50.125869 | -5.524697 | 25 | 0 | 0 | 25 | 0 |
| Crowlas | CRO | England | Land's End | LANDSEND | 50.125802 | -5.480418 | 25 | 0 | 0 | 25 | 0 |
| Tresillian | TRE | England | Devon & Cornwall | DEVCORN | 50.142221 | -5.029527 | 30 | 0 | 0 | 30 | 0 |
| Fal | FAL | England | Devon & Cornwall | DEVCORN | 50.142221 | -5.029527 | 30 | 0 | 0 | 30 | 0 |
| Fowey | FOW | England | Devon & Cornwall | DEVCORN | 50.323694 | -4.642354 | 50 | 0 | 0 | 32 | 18 |
| West Looe | WLO | England | Devon & Cornwall | DEVCORN | 50.350145 | -4.44909 | 30 | 0 | 0 | 30 | 0 |
| East Looe | ELO | England | Devon & Cornwall | DEVCORN | 50.350145 | -4.44909 | 40 | 0 | 1 | 29 | 10 |
| Seaton | SEA | England | Devon & Cornwall | DEVCORN | 50.362856 | -4.388246 | 33 | 0 | 1 | 32 | 0 |
| Lynher | LYN | England | Devon & Cornwall | DEVCORN | 50.32216 | -4.152776 | 30 | 0 | 1 | 29 | 0 |
| Tamar | TAM | England | Devon & Cornwall | DEVCORN | 50.32216 | -4.152776 | 56 | 0 | 1 | 36 | 19 |
| Tavy | TAV | England | Devon & Cornwall | DEVCORN | 50.32216 | -4.152776 | 30 | 0 | 1 | 29 | 0 |
| Plym | PLY | England | Devon & Cornwall | DEVCORN | 50.32216 | -4.152776 | 30 | 0 | 1 | 29 | 0 |
| Yealm | YEA | England | Devon & Cornwall | DEVCORN | 50.300346 | -3.954329 | 30 | 0 | 0 | 30 | 0 |
| Erme | ERM | England | Devon & Cornwall | DEVCORN | 50.310927 | -4.073074 | 59 | 0 | 0 | 37 | 22 |
| Devon Avon | DAV | England | Devon & Cornwall | DEVCORN | 50.276325 | -3.88919 | 51 | 1 | 1 | 28 | 21 |
| Dart | DAR | England | Devon & Cornwall | DEVCORN | 50.333009 | -3.555909 | 72 | 0 | 0 | 42 | 30 |
| Teign | TEI | England | Devon & Cornwall | DEVCORN | 50.539001 | -3.492309 | 38 | 0 | 0 | 30 | 8 |
| Exe | EXE | England | Devon & Cornwall | DEVCORN | 50.608556 | -3.41592 | 61 | 0 | 0 | 32 | 29 |
| Otter | OTT | England | Devon & Cornwall | DEVCORN | 50.627911 | -3.303624 | 30 | 0 | 0 | 30 | 0 |
| Axe | AXE | England | Devon & Cornwall | DEVCORN | 50.701426 | -3.055595 | 30 | 0 | 1 | 29 | 0 |
| Brit | BRI | England | Hampshire Basin | HANTS | 50.707775 | -2.764488 | 30 | 0 | 1 | 29 | 0 |
| Frome | FRO | England | Hampshire Basin | HANTS | 50.677329 | -1.936547 | 78 | 0 | 0 | 48 | 30 |
| Piddle | PID | England | Hampshire Basin | HANTS | 50.677329 | -1.936547 | 30 | 0 | 0 | 30 | 0 |
| Dorset Stour | DST | England | Hampshire Basin | HANTS | 50.721962 | -1.735832 | 30 | 0 | 0 | 30 | 0 |
| Hampshire Avon | HAV | England | Hampshire Basin | HANTS | 50.721962 | -1.735832 | 46 | 0 | 0 | 30 | 16 |
| Lymington | LYM | England | Hampshire Basin | HANTS | 50.741284 | -1.512443 | 42 | 0 | 0 | 30 | 12 |
| Beaulieu | BEA | England | Hampshire Basin | HANTS | 50.78117 | -1.374305 | 30 | 1 | 2 | 27 | 0 |
| Itchen | ITC | England | Hampshire Basin | HANTS | 50.86682 | -1.366846 | 30 | 0 | 0 | 30 | 0 |
| Test | TES | England | Hampshire Basin | HANTS | 50.86682 | -1.366846 | 42 | 5 | 0 | 25 | 12 |
| Hamble | HAM | England | Hampshire Basin | HANTS | 50.843933 | -1.314478 | 30 | 5 | 0 | 25 | 0 |
| Meon | MEO | England | Hampshire Basin | HANTS | 50.816412 | -1.241401 | 30 | 1 | 0 | 29 | 0 |
| Wallington | WAL | England | Hampshire Basin | HANTS | 50.775205 | -1.108099 | 30 | 0 | 0 | 30 | 0 |
| Eastern Yar | YAR | England | South east England | SEENG | 50.69769 | -1.094026 | 30 | 0 | 0 | 30 | 0 |
| Arun | ARU | England | South east England | SEENG | 50.798915 | -0.540928 | 29 | 0 | 0 | 29 | 0 |
| Adur | ADU | England | South east England | SEENG | 50.823797 | -0.247195 | 30 | 0 | 0 | 30 | 0 |
| Sussex Ouse | OUS | England | South east England | SEENG | 50.776616 | 0.060959 | 42 | 0 | 2 | 31 | 9 |
| Eastern Rother | ROT | England | South east England | SEENG | 50.925445 | 0.777134 | 40 | 0 | 0 | 30 | 10 |
| Dour | DOU | England | South east England | SEENG | 51.111552 | 1.329468 | 30 | 0 | 0 | 30 | 0 |
| Great Stour | GST | England | South east England | SEENG | 51.318806 | 1.383149 | 30 | 7 | 0 | 23 | 0 |
| Medway | MED | England | Thames & East Anglia | THAMESEA | 51.462076 | 0.745395 | 30 | 0 | 1 | 29 | 0 |
| Thames | THA | England | Thames & East Anglia | THAMESEA | 51.485119 | 0.795208 | 34 | 7 | 4 | 23 | 0 |
| Belstead Brook | BEL | England | Thames & East Anglia | THAMESEA | 51.896767 | 1.358933 | 30 | 10 | 0 | 20 | 0 |
| Wensum | WEN | England | Thames & East Anglia | THAMESEA | 52.572361 | 1.745058 | 25 | 1 | 1 | 23 | 0 |
| Glaven | GLA | England | Thames & East Anglia | THAMESEA | 52.983507 | 0.96992 | 35 | 2 | 0 | 33 | 0 |
| Nar | NAR | England | Thames & East Anglia | THAMESEA | 52.835396 | 0.341324 | 20 | 0 | 1 | 19 | 0 |
| Yorkshire Esk | ESK | England | North east England | NEENG | 54.493882 | -0.611669 | 16 | 0 | 0 | 16 | 0 |
| Tyne | TYN | England | North east England | NEENG | 55.010573 | -1.394313 | 52 | 0 | 0 | 32 | 20 |
| Coquet | COQ | England | North east England | NEENG | 55.339596 | -1.567247 | 22 | 2 | 0 | 20 | 0 |
| Tweed | TWEE | England | North east England | NEENG | 55.763122 | -1.981566 | 24 | 0 | 0 | 24 | 0 |
| Aber Wrac'h | ABE | France | Bretagne | BRET | 48.610399 | -4.582648 | 26 | 1 | 0 | 25 | 0 |
| Flèche | FLE | France | Bretagne | BRET | 48.644957 | -4.299911 | 31 | 2 | 0 | 29 | 0 |
| Kérallé | KER | France | Bretagne | BRET | 48.657244 | -4.228475 | 32 | 7 | 0 | 25 | 0 |
| Guillec | GUI | France | Bretagne | BRET | 48.688319 | -4.073144 | 31 | 4 | 1 | 26 | 0 |
| Horn | HOR | France | Bretagne | BRET | 48.688681 | -4.059893 | 32 | 0 | 2 | 30 | 0 |
| Yar | FYA | France | Bretagne | BRET | 48.674915 | -3.583281 | 32 | 3 | 1 | 28 | 0 |
| Léguer | LEG | France | Bretagne | BRET | 48.735102 | -3.560884 | 31 | 0 | 0 | 31 | 0 |
| Ic | IC | France | Bretagne | BRET | 48.600973 | -2.814563 | 31 | 1 | 0 | 30 | 0 |
| Frémur | FRE | France | Bretagne | BRET | 48.621228 | -2.143971 | 28 | 0 | 0 | 28 | 0 |
| Couesnon | COU | France | Bretagne | BRET | 48.637091 | -1.51632 | 35 | 3 | 0 | 32 | 0 |
| Selune | SEL | France | Bretagne | BRET | 48.648012 | -1.447503 | 33 | 8 | 1 | 24 | 0 |
| Sée | SEE | France | Bretagne | BRET | 48.648012 | -1.447503 | 35 | 4 | 0 | 25 | 6 |
| Sienne | SIE | France | Bretagne | BRET | 48.990324 | -1.569532 | 34 | 2 | 0 | 32 | 0 |
| Saire | SAI | France | Bretagne | BRET | 49.609818 | -1.250799 | 30 | 0 | 0 | 30 | 0 |
| Vire | VIR | France | Lower Normandie | LNO | 49.365329 | -1.129752 | 31 | 2 | 0 | 29 | 0 |
| Seulles | SEU | France | Lower Normandie | LNO | 49.337897 | -0.456177 | 40 | 3 | 1 | 27 | 9 |
| Orne | ORN | France | Lower Normandie | LNO | 49.293151 | -0.246018 | 33 | 5 | 1 | 27 | 0 |
| Dives | DIV | France | Lower Normandie | LNO | 49.306222 | -0.095141 | 36 | 3 | 0 | 33 | 0 |
| Touques | TOU | France | Lower Normandie | LNO | 49.371122 | 0.068543 | 33 | 0 | 12 | 21 | 0 |
| Seine | SEI | France | Lower Normandie | LNO | 49.431671 | 0.084352 | 60 | 4 | 2 | 30 | 24 |
| Valmont | VAL | France | Upper Normandie | UNO | 49.765316 | 0.362524 | 30 | 3 | 3 | 24 | 0 |
| Durdent | DUR | France | Upper Normandie | UNO | 49.856446 | 0.607286 | 26 | 0 | 0 | 26 | 0 |
| Saâne | SAA | France | Upper Normandie | UNO | 49.906918 | 0.929678 | 30 | 0 | 1 | 29 | 0 |
| Scie | SCI | France | Upper Normandie | UNO | 49.918092 | 1.031633 | 28 | 0 | 0 | 28 | 0 |
| Arques | ARQ | France | Upper Normandie | UNO | 49.936621 | 1.082478 | 76 | 1 | 2 | 40 | 33 |
| Yères | YER | France | Upper Normandie | UNO | 50.033002 | 1.310304 | 27 | 0 | 0 | 27 | 0 |
| Bresle | BRE | France | Upper Normandie | UNO | 50.066402 | 1.368578 | 67 | 0 | 5 | 36 | 26 |
| Somme | SOM | France | Upper Normandie | UNO | 50.23594 | 1.523448 | 31 | 1 | 3 | 27 | 0 |
| Authie | AUT | France | Upper Normandie | UNO | 50.374055 | 1.566111 | 14 | 0 | 0 | 14 | 0 |
| Canche | CAN | France | Upper Normandie | UNO | 50.548323 | 1.578664 | 27 | 2 | 6 | 19 | 0 |
| Liane | LIA | France | Upper Normandie | UNO | 50.737078 | 1.580767 | 34 | 1 | 0 | 33 | 0 |
| Slack | SLA | France | Upper Normandie | UNO | 50.805 | 1.600931 | 33 | 4 | 0 | 29 | 0 |
| Sneum | SNE | Denmark | Denmark | DENMARK | 55.905366 | 8.355854 | 40 | 0 | 2 | 38 | 0 |
| Skjern | SKJ | Denmark | Denmark | DENMARK | 55.361061 | 8.334486 | 29 | 0 | 0 | 29 | 0 |
| Hatchery J | HATJ | Hatchery | French hatchery | FRHAT | - | - | 30 | 0 | 0 | 30 | 0 |
| Hatchery PF | HATPF | Hatchery | French hatchery | FRHAT | - | - | 26 | 0 | 0 | 26 | 0 |
| Total |  |  |  |  |  |  | 3699 | 125 | 71 | 3067 | 436 |
