# Supplementary material for: Leveraging the genetic diversity of trout in the rivers of the British Isles and northern France to understand the movements of sea trout (Salmo trutta L.) around the English Channel
Source: Evol Appl. 2024 Jul 22;17(7):e13759. doi: 10.1111/eva.13759 (PMC11261213; doi:10.1111/eva.13759)
Supplement: Supplementary file 8 — Table S6 [file EVA-17-e13759-s008.docx]

**Supplementary Table 6 for:**

**Leveraging the genetic diversity of trout in the rivers of the southern British Isles and northern France to understand the movements of sea trout (*Salmo trutta* L.) around the English Channel**

**Supplementary Table 6** Least cost distances between marine and estuarine sampling locations and rivers in each reporting group. Distances were calculated using the marmap R package (Pante & Simon-Bouhet, 2013). Min – minimum distance between sea trout sampling location and a river in a reporting group; max - maximum distance between sea trout sampling location and a river in a reporting group; average - average distance between sea trout sampling location and each river in a reporting group. Distances are presented only for the reporting groups to which sea trout from each marine or estuarine collection were assigned, based on Individual Assignment results (Supplementary Tables 5.01-5.12).

|  |  | **Marine** | | | | | | | | **Estuarine** | | | |
| --- | --- | --- | --- | --- | --- | --- | --- | --- | --- | --- | --- | --- | --- |
| **Reporting Group** | **Distance** | **COR** | **KIM** | **RYE** | **EAN** | **SAA** | **CRI** | **MER** | **DUT** | **TT** | **TAM** | **PLH** | **OUS** |
| OUTBRCH | min | 288 | - | - | - | - | - | - | - | 0 | - | - | - |
|  | max | 363 | - | - | - | - | - | - | - | 92 | - | - | - |
|  | average | 330.3 | - | - | - | - | - | - | - | 61.3 | - | - | - |
| DEVCORN | min | 7 | 76 | - | - | - | - | - | - | 81 | 0 | - | - |
|  | max | 209 | 372 | - | - | - | - | - | - | 397 | 215 | - | - |
|  | average | 71.9 | 176.7 | - | - | - | - | - | - | 287.1 | 70.7 | 197.9 | - |
| HANTS | min | - | 30 | 144 | 418 | - | - | - | - | - | - | 0 | - |
|  | max | - | 86 | 272 | 546 | - | - | - | - | - | - | 77 | - |
|  | average | - | 63.6 | 181.3 | 455.1 | - | - | - | - | - | - | 44.6 | - |
| SEENG | min | - | 90 | 6 | - | - | - | - | - | - | - | - | 0 |
|  | max | - | 271 | 143 | - | - | - | - | - | - | - | - | 107 |
|  | average | - | 172.0 | 73.7 | - | - | - | - | - | - | - | - | 53.8 |
| NEENG | min | 879 | 715 | 491 | 225 | - | - | - | 515 | - | - | - | - |
|  | max | 1040 | 876 | 652 | 386 | - | - | - | 640 | - | - | - | - |
|  | average | 964.8 | 800.8 | 576.3 | 310.3 | - | - | - | 583.5 | - | - | - | - |
| BRET | min | - | 140 | - | - | - | - | - | - | - | - | - | - |
|  | max | - | 292 | - | - | - | - | - | - | - | - | - | - |
|  | average | - | 238.0 | - | - | - | - | - | - | - | - | - | - |
| LOWNORM | min | - | 161 | - | - | - | - | - | - | - | - | - | - |
|  | max | - | 215 | - | - | - | - | - | - | - | - | - | - |
|  | average | - | 199.2 | - | - | - | - | - | - | - | - | - | - |
| UPPNORM | min | - | - | - | - | 0 | 0 | 0 | 500 | - | - | - | - |
|  | max | - | - | - | - | 116 | 94 | 91 | 644 | - | - | - | - |
|  | average | - | - | - | - | 53.8 | 48.6 | 49.1 | 575.6 | - | - | - | - |
| DENMARK | min | - | - | - | - | - | - | - | 215 | - | - | - | - |
|  | max | - | - | - | - | - | - | - | 278 | - | - | - | - |
|  | average | - | - | - | - | - | - | - | 246.5 | - | - | - | - |
